# High-Quality Epitaxial N Doped Graphene on SiC with Tunable Interfacial Interactions via Electron/Ion Bridges for Stable Lithium-Ion Storage

**DOI:** 10.1007/s40820-023-01175-6

**Published:** 2023-08-18

**Authors:** Changlong Sun, Xin Xu, Cenlin Gui, Fuzhou Chen, Yian Wang, Shengzhou Chen, Minhua Shao, Jiahai Wang

**Affiliations:** 1 School of Chemistry and Chemical Engineering, Guangzhou University, Guangzhou, 510006 People’s Republic of China; 2 Department of Chemical and Biological Engineering, The Hong Kong University of Science and Technology, Clear Water Bay, Kowloon, Hong Kong People’s Republic of China

**Keywords:** SiC, Heterojunction, Interfacial engineering, Lithium-ion battery, DFT calculation

## Abstract

Tailoring the interfacial interaction in SiC-based anode materials is crucial to the accomplishment of higher energy capacities and longer cycle lives for lithium-ion storage. In this paper, atomic-scale tunable interfacial interaction is achieved by epitaxial growth of high-quality N doped graphene (NG) on SiC (NG@SiC). This well-designed NG@SiC heterojunction demonstrates an intrinsic electric field with intensive interfacial interaction, making it an ideal prototype to thoroughly understand the configurations of electron/ion bridges and the mechanisms of interatomic electron migration. Both density functional theory (DFT) analysis and electrochemical kinetic analysis reveal that these intriguing electron/ion bridges can control and tailor the interfacial interaction via the interfacial coupled chemical bonds, enhancing the interfacial charge transfer kinetics and preventing pulverization/aggregation. As a proof-of-concept study, this well-designed NG@SiC anode shows good reversible capacity (1197.5 mAh g^−1^ after 200 cycles at 0.1 A g^−1^) and cycling durability with 76.6% capacity retention at 447.8 mAh g^−1^ after 1000 cycles at 10.0 A g^−1^. As expected, the lithium-ion full cell (LiFePO_4_/C//NG@SiC) shows superior rate capability and cycling stability. This interfacial interaction tailoring strategy via epitaxial growth method provides new opportunities for traditional SiC-based anodes to achieve high-performance lithium-ion storage and beyond.

**Supplementary Information:**

The online version contains supplementary material available at 10.1007/s40820-023-01175-6.

## Highlights

- The intimate NG@SiC heterostructure has been constructed via a direct thermal decomposition method.
- The NG@SiC heterostructure anode delivers enhanced capacity and cycling stability both in the half-cell and in the full cell.
- DFT analysis reveals that this NG@SiC anode possesses lower lithium-ion adsorption energy and higher charge and discharge rates.

**Supplementary Information:**

The online version contains supplementary material available at 10.1007/s40820-023-01175-6.

## Graphical abstract

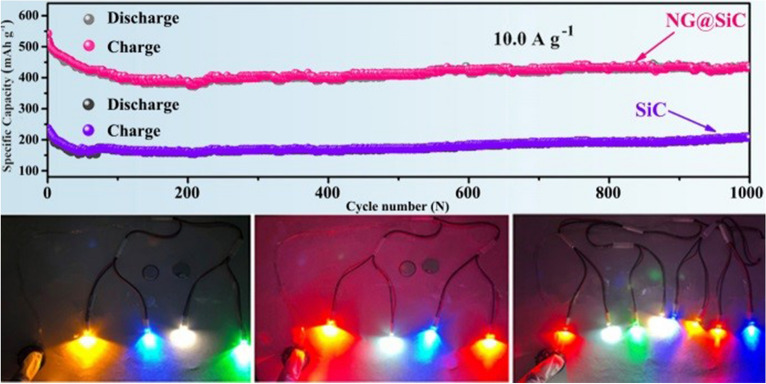

## Introduction

As widely used energy storage devices, lithium-ion batteries (LIBs) have attracted extensive attention for storing energy generated from hydropower, wind, and solar [1]. Since commercial graphite acting as the active materials for LIB anodes shows unsatisfactory theoretical capacity and insufficient high-rate capability [2], some alternative anode-active materials with high capacities, long cycle lives, and short charging times have been extensively researched in recent years [3]. Among different types of LIB anode materials, Si-based nanostructured anodes have been recently researched as feasible alternatives owing to their advantages such as higher charge storage capacities and resource abundance [4]. Despite the tremendous progress, lithiated Si-based anodes still suffer from drastic volume changes (> 300%) caused by continuous lithiation and delithiation reactions, which can lead to fast pulverization, poor rate performance and charge storage fading [5]. Among these Si-based anodes, silicon carbide (SiC), which is usually perceived to be electrochemically inert for lithium-ion storage, attracts great attention owing to the excellent structural stability, lower conversion potential. Specially, lithium-ion could be mainly occupied on the interstitial sites at (1/4, 1/4, 1/4) and (1/2, 1/2, 1/2) of SiC with tetrahedral symmetry, providing the possibilities for electrochemical lithiation of SiC in LIBs, and the total number of Li intercalated in SiC is 12, so that it can form the Li_2_SiC composition for which the specific capacity is calculated to be 1336 mAh g^−1^ [6]. Therefore, SiC-based materials are considered to be promising alternative anode materials with outstanding chemical stability during electrochemical reactions [7]. However, SiC is usually used as a structural reinforcement layer to improve the stability of conventional anode-active materials [8–10]. According to a previous report, the interstitial sites of SiC lattices can be occupied by lithium ions and activated for lithium-ion insertion via structural design and surface modification [11]. As for practical applications, the unsatisfied electronic conductivity and ion diffusion of SiC-based materials can result in the low efficiency and inferior performance, which restrict the rate capability and cycle capacity of SiC-based anodes [12]. Furthermore, the re-formed solid-electrolyte interphase (SEI) film during the electrochemical reaction could easily lead to inferior Coulombic efficiency and “dead lithium” near the SiC surface [13].

To address the above issues associated with SiC-based anodes, two strategies, morphological modification and structural optimization, have been adopted to enhance the lithium-ion storage performance [14, 15]. However, the resulting electrochemical properties are far inferior to those of conventional anode-active materials, and the intrinsic improvement of the charge transfer kinetics has not been achieved. Thus, the rate capability and cycle capacity are still unsatisfactory and need to be improved. Recently, Kumari et al. confirmed that nanoscopic carbon coated SiC demonstrated extended cyclability with negligible structural change during lithium-ion insertion and desertion [16]. Li et al. designed ultrathin SiC nanoshells in hollow carbon spheres, and the well-designed SiC anode exhibited high-battery capacities, which can be attributed to the deliberate structural design with good durability [17]. Despite all these breakthroughs, the electronic conductivity of conventional carbon sphere is much lower than that of graphene, and it is unclear whether interfacial interactions exist in the SiC-based anodes. Therefore, the currently achievable electrochemical properties are much inferior to those of the well-known anode-active materials. As a result, the applications of pristine SiC anodes are limited in energy storage fields. It is interesting that surface graphitization can convert SiC from the electrochemically inert state to the electrochemically active state and can accelerate the intercalation of lithium ions into SiC lattices with enhanced electronic conductivity and electrochemical capacity [18]. Moreover, the electric field of SiC should be generated at the interface via the strong bridging bond, according to the energy band theory [19]. In this way, the electron/ion bridge would be a potential charge transfer channel to enhance the electrochemical performance. Nonetheless, the construction of sufficient electron/ion bridges in SiC-based materials has rarely been investigated up to date, and the fundamental mechanism of lithium-ion storage that leads to enhanced performance remains unclear. Hence, *in situ* epitaxial high-quality nitrogen-doped graphene (NG) on SiC (NG@SiC) particles with tunable interfacial interactions appear feasible to construct strong electron/ion bridges to improve the battery capacities of SiC-based anodes.

In this contribution, the interfacial interactions at the NG@SiC heterojunction are constructed and controlled via the delicately designed interfacial bridging bonds (C–Si–C), opening an extra electron/ion transport channel to enhance the interfacial charge transfer kinetics and to prevent pulverization or aggregation during cycling. Density functional theory (DFT) analysis reveals that such an NG@SiC composite anode possesses a lower lithium-ion adsorption energy than the pristine SiC anode. The bridging bonds formed at the heterointerface can act as an electron/ion transport channel to accelerate the reaction kinetics and to improve the lithium-ion storage performance, which is confirmed by the electrochemical and kinetic analysis. The excellent full cell performance shows the practicability of the NG@SiC composite anode. This is the first time that *in situ* epitaxial high-quality nitrogen-doped graphene is used to reveal the relationship between interfacial interactions and lithium-ion storage of SiC-based anodes, and the strategy of creating interfacial bridging bonds provides a promising way to re-design traditional electrode materials.

## Experimental Section

### NG@ SiC Preparation

All chemical reagents are analytically pure and purchased from Aladdin Chemical Co. Ltd, China. The raw SiC particles were ultrasonically washed with pure ethanol and deionized water for 30 min. Then, the SiC powder was heated to 100 ºC in a bake oven for overnight to remove the absorbed water. Then, the SiC particles was transferred to a graphite crucible and loaded into a tube furnace. This reaction process was performed in the chemical vapor deposition reactor. The quartz reactor was pumped to the base pressure (≤ 1 mTorr) by a mechanical dry pump and then, purged with argon before the growth. The high-quality epitaxial NG was grown on SiC particles by high-temperature annealing in ammonia atmosphere at 1500 °C for 15 min. After high-temperature annealing, the epitaxial NG layer can be directly formed by sublimation of silicon atoms from surface of SiC particles, and this is a quasi-freestanding epitaxial process. During this pyrolysis reaction, the high-quality epitaxial NG layer is formed after atomic nitrogen intercalates into the as-formed epitaxial graphene. At the end of the growth, the tube furnace was cooled down naturally to room temperature.

### Characterization Methods

The structure and morphology of pristine SiC and NG@SiC are characterized by scanning electron microscopy (SEM, Hitachi S-4800). The EDX elemental spectroscopy of pristine SiC and NG@SiC are observed using a Talos TEM (FEI, Talos F200X). The powder X-ray diffraction (XRD) measurements were performed on a Rigaku D/MAXRB diffractometer from 20° to 80° (Philips, X’pert Pro MPD, Netherlands). Transmission electron microscopy (TEM) images are obtained using a Philips Tecnai 20U-Twin microscope at an acceleration voltage of 200 kV. Raman spectra are measured with 532 nm photons. The binding energy is acquired by X-ray photoelectron spectroscopy (XPS) on the Thermo ESCALAB 250 with Al Kα radiation (1486.8 eV) as the excitation source. For the X-ray absorption near edge structure (XANES), Si *K*-edge spectrum and C *K*-edge spectrum are collected at Beijing Synchrotron Radiation Facility (BSRF).

### Electrochemical Measurements

CR2016-type coin cells are used to investigate the electrochemical performance of pristine SiC and NG@SiC. 80 wt% active materials, 10 wt% conductive carbon (ketjen black), and 10 wt% polyvinylidene fluoride (PVDF) as the binder were mixed in N-methyl-2-pyrrolidone; then, the mixture is pasted on copper foil and dried at 80 °C. Li metal is used as both the counter and reference electrodes. Li metal cathode and NG@SiC anode are electronically separated by a polypropylene film (Celgard 2320) saturated with electrolyte. The electrolyte solution is consisting of LiPF_6_ (1 M) in ethylene carbonate/dimethyl carbonate/diethyl carbonate (1:1:1 vol%). Neware CT-3008W system (Neware Technology Ltd., P. R. China) is used to carry out galvanostatic charge and discharge tests at various current densities. The galvanostatic intermittent titration technique (GITT) is tested on CT-3008W system as well. The cell was discharged at 0.5 A g^‒1^ for 5 min, followed by a 20 min relaxing from 0.01 to 3.0 V. The cyclic voltammetry (CV) is performed at different scan rate from 0.01 to 3.0 V via the CHI760E electrochemical workstation (Shanghai CH Instruments Co., China). Electrochemical impedance spectroscopy (EIS) test is also recorded by CHI760E electrochemical workstation with the frequency ranging from 0.01 Hz to 1 MHz at a 5 mV amplitude signal without applied voltage bias.

### Li-ion Full Cell (LiFePO_4_/C//NG@SiC) Preparation

The LiFePO_4_/C//NG@SiC full cell is fabricated using the commercial LiFePO_4_/C cathode (composed of 70 wt% LiFePO_4_/C, 20 wt% carbon black, and 10 wt% PVDF) and NG@SiC anode. The processes of LiFePO_4_/C//NG@SiC full cells preparation are similar to that of half-cells, and Al foils is used as the current collector. For LiFePO_4_/C//NG@SiC full cell, the NG@SiC anode is first discharged to 0.01 V at 0.1 A g^‒1^ in half-cell to compensate the loss of lithium during the initial cycle. The LiFePO_4_/C//NG@SiC full cell is charged/discharged at a voltage range of 1.0 − 4.0 V. In order to alleviate the formation of lithium dendrites during cycling, the capacity ratios of anode and cathode are controlled around 1.1: 1. The calculation process is as follows:

$$\frac{{C_{{{\text{anode}}}} \times m_{{{\text{anode}}}} }}{{C_{{{\text{cathode}}}} \times m_{{{\text{cathode}}}} }} = \, 1.1 \, : \, 1$$

where *C* is the specific capacity, and *m* is the loading mass. The mass loading of NG@SiC anode is 2.0 mg. At 0.1 A g^−1^, *C*_anode_ is 1197.5 mAh g^−1^, and *C*_cathode_ is 152.4 mAh g^−1^. Therefore, the mass loading of the LiFePO_4_ cathode is 14.2 mg, and the calculated mass ratio of anode and cathode was fixed at 1: 7.1. The specific capacity of the LiFePO_4_/C//NG@SiC full cell is calculated based on the total weight of the active materials (LiFePO_4_/C cathode and NG@SiC anode).

### Density Functional Theory Calculation

The calculations were based on the DFT combined with the projector-augmented-wave potential as implemented by the Vienna ab initio simulation package. The generalized gradient approximation (GGA) exchange–correlation function developed by Perdew, Burke and Ernzerhof was used for the exchange–correlation potential. The cutoff energy for plane-wave expansion was set to 440 eV. All the SiC-based structures were relaxed until the forces became less than 0.01 eV Å^−1^, and the energy tolerances were less than 10^−5^ eV atom^−1^. The DFT + U method was used with the Dudarev approach implemented in Vienna ab initio Simulation Package, where U is the on-site Coulomb parameter to calculate the average voltages of Li intercalation. The K-point of the Brillouin zone was sampled using 3 × 3 × 1 gamma-centered Monkhorst–Pack grid for the unit cell. A vacuum of 20 Å between the layers was considered to safely avoid the interaction between the periodically repeated structures. We used the climbing image nudged elastic band method (CI-NEB) to determine the energy barriers and minimum energy paths of Li diffusion. The Li adsorption was studied to demonstrate theoretical proof for the electrocatalytic performance. Li adsorption energies were defined as *ΔE* = *E*_slab+Li_–*E*_slab_–*E*_Li_. Each transition state showed only one imaginary frequency, and vibration mode displayed the right path connecting the reactant and product.

## Results and Discussion

### Structural Design and Preparation of NG@SiC

**Fig. 1:**
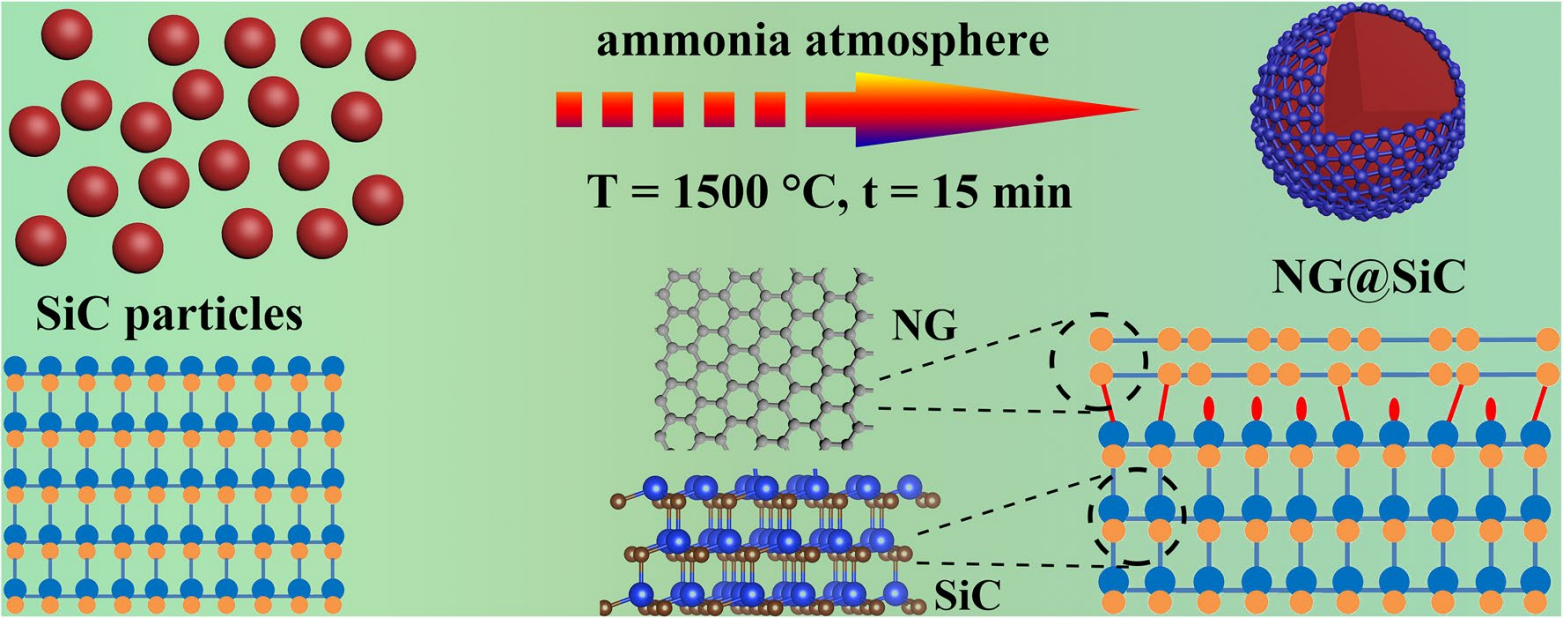
Schematic illustration of synthetic process of NG@SiC

As shown in Fig. 1, the high-quality epitaxial NG was grown on SiC particles by high-temperature annealing in ammonia atmosphere, which is a facile and repeatable strategy. This reaction process was performed in a chemical vapor deposition reactor in a quartz tube furnace. After high-temperature annealing, the epitaxial NG layer can be directly formed by sublimation of silicon atoms from the surface of SiC particles via a quasi-freestanding epitaxial process [20]. During this pyrolysis reaction, the high-quality epitaxial NG layer was formed after the atomic nitrogen intercalated into the as-formed epitaxial graphene. Unlike graphitic carbon, the high-quality epitaxial NG layer shows a higher electronic conductivity [21] and grows with disorder under ammonia atmosphere [22]. This interfacial layer is considered to be zero-layer NG, which consists of graphene-like honeycomb carbon atoms with atomic displacements because of the covalent interlayer bonds formed with the SiC particles. Moreover, the interatomic electron migration was formed owing to the interfacial layer at the NG@SiC heterojunction. After high-temperature annealing in ammonia atmosphere, the covalent interlayer bonds in the interfacial NG layer were passivated, which can act as electron/ion bridges to enhance the interfacial charge transfer kinetics and to prevent pulverization or aggregation during cycling. After the reconstruction process, the outer NG layer was deposited on the interfacial NG layer without forming covalent bonds with SiC, whose electrical properties are close to those of theoretical graphene. Although the covalent bonding inhibits the mobility of the interfacial NG layer, it can convert SiC from the electrochemically inert state to the electrochemically active state with enhanced electronic conductivity. Moreover, the interatomic electron migration and electrochemical reaction kinetics are highly dependent on the covalent interlayer bonds between the interfacial NG layer and SiC particles.

### Structural Characterizations

**Fig. 2:**
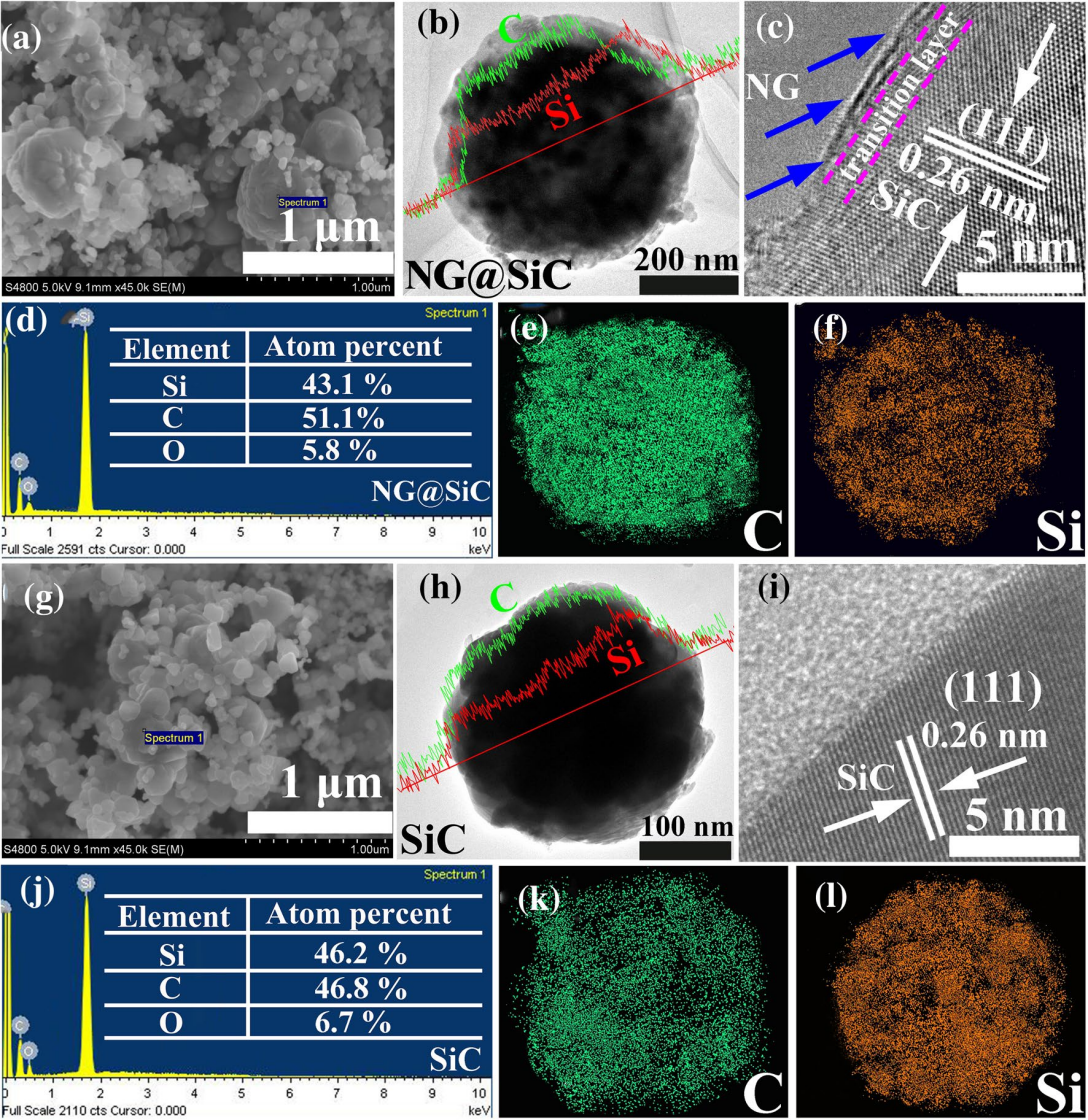
**a** SEM, **b** TEM (inset: linear elemental scan analysis), and **c** HRTEM images of NG@SiC. **d-f** EDX and corresponding elemental-mapping analysis of NG@SiC. **g** SEM, **h** TEM (inset: linear elemental scan analysis), and **i** HRTEM images of pristine SiC. **j-l** EDX and corresponding elemental-mapping analysis of pristine SiC

As shown in the SEM image (Fig. 2a), after high-temperature annealing in ammonia atmosphere, the size of NG@SiC particles is about 500 nm. Compared with the pristine SiC (Fig. 2g), the larger size of NG@SiC particles can be attributed to the aggregation of small pristine SiC particles to decrease the surface energy under high-temperature annealing. The lamellar NG layer is clearly visible on the surface of the NG@SiC particle via the low-magnification TEM image (Fig. 2b), and the outer NG layer attaches tightly on the SiC particle with contact interfaces. In contrast, the outer NG layer does not existent on the pristine SiC particle (Fig. 2h). The linear scan elemental analyses indicate the existence of Si and C elements in both NG@SiC and pristine SiC. HRTEM analysis was performed to further demonstrate the existence of the high-quality epitaxial NG layer. In Fig. 2c, it can be clearly observed that the outer NG layer is tightly coated on the surface of NG@SiC particles with an interlayer spacing of about 0.35 nm, and the interfacial NG layer (transition layer in Fig. 2c) exists between the outer NG layer and the SiC particles, which can act as the electron/ion bridge to enhance the interfacial charge transfer kinetics. For the inner SiC layer, the measured inter-planar spacing is about 0.26 nm, corresponding to the (111) lattice spacing of SiC. In contrast, the outer NG layer does not exist on the surface of the pristine SiC particles (Fig. 2i), and the (111) lattice spacing of about 0.26 nm indicates that the NG layer has no influence on the crystallinity of SiC particles. Therefore, the epitaxial NG layer is directly deposited on the surface of SiC particles after sublimation of silicon atoms under high-temperature annealing. In order to decrease the surface energy, the NG@SiC particles under thermodynamics could become larger through the merging of small particles, and the surface of the NG@SiC particles would undergo a significant modification after NG layer growth, resulting in the increase of particle size. The corresponding growth mechanism of NG@SiC particle is traditional sintering. The EDX elemental content analyses (Fig. 2d, j) show that the atomic ratios of Si to C elements are approximately 1: 1 for the pristine SiC and 1: 1.18 for NG@SiC. After the epitaxial NG growth, the content of C element increases, while that of O element decreases. This result reveals that the outer NG layer can protect the NG@SiC particles from oxidation, and the outer NG layer is successfully deposited by sublimation of silicon atoms from the surface of SiC particles. The corresponding EDX elemental-mapping results (Fig. 2e, f and k, l) reveal the uniform distribution of Si and C elements in both NG@SiC and pristine SiC. However, the NG@SiC and pristine SiC particles cannot be clearly distinguished from the linear scan results and the corresponding EDX elemental mapping and content results.

**Fig. 3:**
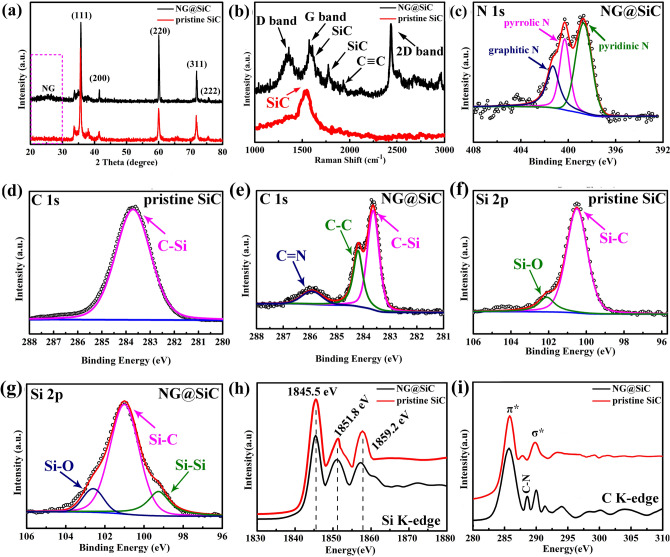
**a** XRD and **b** Raman of pristine SiC and NG@SiC, respectively. XPS spectra: **c** N 1*s* of NG@SiC. **d, e** C 1*s* and **f, g** Si 2*p* of pristine SiC and NG@SiC, respectively. **h, i** Si *K*-edge and C *K*-edge XANES spectra of pristine SiC and NG@SiC

In Fig. 3a, the XRD analysis reveals that all diffraction peaks of pristine SiC and NG@SiC correspond to SiC (JCPDS No. 29–1129). The typical (111), (220), and (311) diffraction peaks can be clearly observed in both pristine SiC and NG@SiC, showing that the NG layer has no influence on the crystallinity of NG@SiC particles. For NG@SiC, the tiny peak at ~ 22° corresponds to the NG layer, which is not observed in the pristine SiC. This result indicates that the epitaxial NG layer is directly grown on the surface of NG@SiC particles after sublimation of silicon atoms under high-temperature annealing in ammonia atmosphere [23]. To further confirm the existence of the epitaxial NG layer, Raman analysis was performed. As shown in Fig. 3b, the Raman peaks of SiC are observed in both pristine SiC and NG@SiC [24]. The G and D peaks represent the in-phase stretching vibration and the breathing vibration of sp^2^ carbon, respectively. The D, G, and 2D peaks can be well identified as the typical characteristics of epitaxial NG layer in NG@SiC, while the graphene-related Raman peaks do not exist in the pristine SiC. On the other hand, the band at about 1993 cm^−1^ can be observed in NG@SiC, which corresponds to the C≡C vibration in graphene [25]. As previously reported, the structural defect in graphene can be determined by the intensity ratio of D and G peaks (*I*_D_/*I*_G_), and a lower *I*_D_/*I*_G_ indicates fewer structural defects in graphene [26]. For NG@SiC, the reduced intensity ratio of *I*_D_/*I*_G_ (0.62) manifests the higher crystallinity of epitaxial NG layer, as compared to that of the reduced graphene oxide (*I*_D_/*I*_G_ ≈ 1.0) [27]. After the introduction of nitrogen atoms, the *I*_D_/*I*_G_ value of NG@SiC is higher than that of the reported ideal graphene, indicating the existence of more structural defects in epitaxial NG layer than in ideal graphene. These structural defects can be mainly ascribed to the N-introduced disordered carbon in the epitaxial NG layer [28]. Nonetheless, the nitrogen atoms doped in the epitaxial NG layer not only enhance the electronic conductivity of NG@SiC particles but also provide sufficient active sites for lithium-ion storage. Based on the above analysis, the distinct G, D, 2D, and C≡C peaks of graphene in NG@SiC particles demonstrate the successful preparation of epitaxial NG layer after sublimation of Si atoms under high-temperature annealing, whose crystallinity is nearly the same as that of ideal graphene. Owing to the existence of quasi-freestanding epitaxial NG layer with high crystallinity, the electronic conductivity of NG@SiC particles is dramatically improved, as revealed by the Hall effect analysis. Moreover, the quasi-freestanding epitaxial NG layer with the designed interfacial bridging bonds provides extra electron/ion transport channels to enhance the interfacial charge transfer kinetics during the electrochemical reaction.

To confirm the interfacial bridging bond formation and the chemical composition after the epitaxial NG growth, XPS analysis was performed. In Fig. 3c, the high-resolution N 1*s* spectrum shows three typical peaks, corresponding to graphitic N (401.6 eV), pyrrolic N (400.8 eV), and pyridinic N (399.2 eV), respectively [29]. The ratio of different types of nitrogen in high-resolution N 1*s* spectrum is 1.0:5.6:6.8, and the calculated nitrogen content in the NG@SiC particles is about 3.6%. This result shows that pyridinic N and pyrrolic N are the major nitrogen types in the NG@SiC particles. It is worth noting that the structural defects caused by pyrrolic N and pyridinic N can boost the electronic conductivity of epitaxial NG layer and provide sufficient active sites for lithium-ion storage. In Fig. 3d, the high-resolution C 1*s* spectrum of pristine SiC particles shows the C–Si bond at 283.6 eV [30]. On the other hand, the deconvoluted high-resolution C 1*s* spectrum of NG@SiC (Fig. 3e) shows three distinct peaks at about 283.4, 284.3, and 286.1 eV [31]. The deconvoluted peak at 283.4 eV can assigned to the C–Si bond and that at 284.3 eV corresponds to the *sp*^2^ hybridized carbon (C=C bond), which further confirms the existence of epitaxial NG layer after sublimation of silicon atoms under high-temperature annealing [32]. Moreover, the interfacial NG layer demonstrates strong covalent bonds with the outer NG layer and the SiC particles, which play an important role in the interfacial charge transfer kinetics via the electron/ion bridges. The deconvoluted peak at 286.1 eV can be assigned to the C=N bond in the epitaxial NG layer, showing the nitrogen atom doping in the epitaxial NG layer. It has been reported that nitrogen doping can be used to regulate the electronic properties of graphene [33]. The calculated NG content in NG@SiC is about 3.9% (mass percent). Therefore, NG is conducive to enhancing the electronic conductivity and providing sufficient active sites for lithium-ion storage. In Fig. 3f, the high-resolution Si 2*p* spectrum of pristine SiC particles shows the Si–C and Si–O bonds at 100.4 and 102.2 eV, respectively [34]. For NG@SiC, the high-resolution Si 2*p* spectrum (Fig. 3g) can be deconvoluted to the Si–O, Si–C and Si–Si bonds at 102.5, 100.7, and 99.2 eV, respectively. The distinct Si–Si bond in NG@SiC shows the decomposition of SiC particles with the formation of elemental silicon after sublimation of silicon atoms under high-temperature annealing. According to the XPS results, the elemental silicon concentration is about 1.4 at%. Because of the interfacial NG layer coating, the area ratio of Si–O bond is reduced in NG@SiC particles. Therefore, the XPS analysis further confirms that the quasi-freestanding epitaxial NG layer was successfully deposited on the surface of SiC particles, and the epitaxial NG layer can protect NG@SiC particles from oxidation.

In Fig. 3h, both the pristine SiC and NG@SiC possess similar features of Si *K*-edge X-ray absorption near edge structure (XANES). The peaks at about 1845.5, 1851.8, and 1859.2 eV correspond to the absorption structure of SiC [35]. The typical peak at 1845.5 eV is related to the resonance from Si 1*s* to π*-like hybridized states of Si–C bonds [36]. Moreover, the broader absorption structure of NG@SiC than that of the pristine SiC shows more intensive electronic interactions. Nonetheless, the similar characteristic features reveal that both the pristine SiC and NG@SiC show the same local structures of Si–C bonds, and the NG layer has no influence on the crystallinity of NG@SiC. In Fig. 3i, the C *K*-edge XANES analysis reveals that both the pristine SiC and NG@SiC have rich dipole transitions, which correspond to the electronic interactions from C 1*s* to the unoccupied 2*p* states above the Fermi level [37]. The broader dipole transition of C *K*-edge XANES of NG@SiC shows the enhanced interfacial interaction between the outer epitaxial NG layer and the SiC surface, consistent with the DFT analysis in the following discussion. The peaks at about 285.6 and 290.6 eV correspond to the dipole transitions from C 1*s* to π* and σ*, respectively [37]. The enhanced dipole transition intensity at 285.6 eV can be ascribed to the increased graphite-like *sp*^2^/*sp*^3^ configuration after the deposition of the epitaxial NG layer [38]. The sharper dipole transition peak at 290.6 eV reveals the distinct interfacial interaction between the inner epitaxial NG layer and the SiC surface. The positive peak shift indicates the distinct electron transfer between the epitaxial NG layer and SiC [39]. Compared with the pristine SiC, the dipole transition peak at 294.7 eV shows a different electronic state in NG@SiC [40]. Thus, the dipole transition difference further reveals the intensive interfacial electronic interactions in NG@SiC. In addition, the distinct absorption peak at 288.2 eV corresponds to the C–N bond, revealing the nitrogen doping in the epitaxial NG layer [41]. This XANES result further confirms that the epitaxial NG layer was successfully deposited on the SiC particles after sublimation of silicon atoms under high-temperature annealing in ammonia atmosphere. Owing to the existence of interfacial chemical bonds, NG@SiC shows a high-interfacial electron transfer efficiency, which is crucial for the improved lithium-ion storage performance.

### Electrochemical Performance

**Fig. 4:**
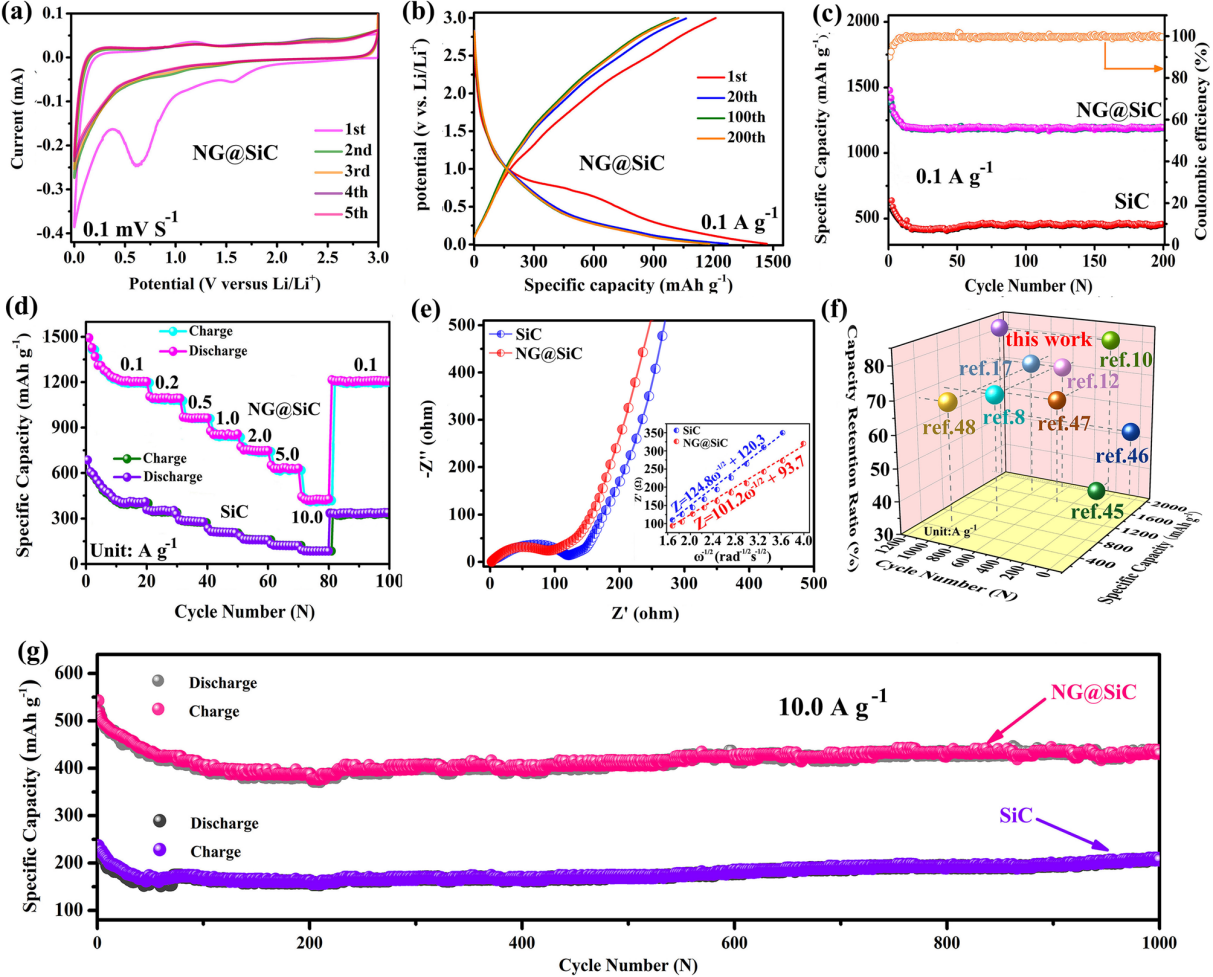
Electrochemical performance of NG@SiC anode: **a** CV at 0.1 mV s^−1^. **b** Galvanostatic charge–discharge (GCD) curves and **c** Cycling property at 0.1 A g^−1^. **d** Rate capabilities and **e** EIS of NG@SiC and SiC. **f** Cycling property comparison with the reported Si-based anodes. **g** Long cycle property at 10.0 A g^−1^

The lithium-ion intercalation and de-intercalation behavior of the NG@SiC anode was investigated by CV curves. Figure 4a shows the CV curves of the NG@SiC anode for the initial 5 cycles at a scan rate of 0.1 mV s^−1^. The CV curves are similar to those of the reported SiC anodes [17], indicating that the epitaxial NG layer can enhance the electrochemical performance but has no substantial effects on the lithium-ion storage mechanism of the SiC anode. The broad peak at around 0.7 V in the first cycle is attributed to the solid-electrolyte-interphase (SEI) film [42], which disappears in the following electrochemical reaction. The initial irreversible steps at around 1.6 V are due to the consumption of lithium by side reaction of NG, SiC, or silicon suboxides with electrolyte occurring in the first cycle, as well as the decomposition of the electrolyte on the partially lithiated electrode. Compared with the pristine SiC anode, the smaller value difference (320 mV) between the lithium-ion intercalation peak and de-intercalation peak of the NG@SiC anode indicates a narrower potential polarization and a faster lithium-ion intercalation kinetics [43]. This result can be attributed to the distinct interfacial interactions and interatomic electron migrations after the epitaxial NG layer growth. The overlapped CV curves and the stabilized lithium-ion intercalation and de-intercalation peaks from the second to fifth CV cycles demonstrate the excellent stability of lithium-ion storage reaction and the structural reversibility of the NG@SiC anode. Besides, the CV curves of the NG@SiC anode are different from those of carbon or silicon anodes, as no formation of Li_x_C or Li_x_Si is observed during the whole electrochemical reaction process [44]. This result shows that no conversion or alloying reaction occurred throughout the discharge/charge process, and the structure of NG@SiC was well preserved during the solid-solution transition. Therefore, the lithium-ion intercalation and de-intercalation mechanism are initially identified with the NG@SiC anode.

 Figure 4b shows the galvanostatic charge–discharge (GCD) curves at 0.1 A g^−1^ of the NG@SiC anode. The initial discharge capacity reaches 1496.8 mAh g^−1^, and the initial charge capacity reaches 1285.4 mAh g^−1^. The corresponding initial coulombic efficiency is 85.8%. The irreversible capacity loss is caused by the decomposition of carbonate electrolyte and the formation of SEI film. Besides, no obvious discharge or charge plateau was detected during the electrochemical reaction, which is different from the GCD curves of carbon or silicon anodes. Compared with the pristine SiC anode, the higher initial coulombic efficiency of the NG@SiC anode can be ascribed to the enhanced interatomic electron migration and charge transfer kinetics after the epitaxial NG layer growth. The nearly overlapped discharge–charge curves also show the excellent reversibility of the lithium-ion storage reaction and the high-structural reversibility of NG@SiC, which is consistent with the CV analysis. Figure 4c shows the cycle performance of the NG@SiC anode at 0.1 A g^−1^. It is important to note that the capacity fading is clearly visible in the first 8 cycles, which is induced by the irreversible lithium-ion consumption. After the initial activation process, the lithium-ion storage reaction becomes stable, and this result is associated with a lithiation-induced reactivation process. In the following cycles, the coulombic efficiency gradually stabilizes at ~ 100% after the first few cycles. In contrast, the specific capacity fading is also visible in the first 16 cycles with the pristine SiC anode, and the specific capacity is much lower than that of the NG@SiC anode. The cycle performance also shows the good electrochemical reversibility of the NG@SiC anode, in good agreement with the CV analysis in Fig. 4a. After 200 cycles, the NG@SiC anode still maintains the excellent reversibility, and the discharge capacity is 1197.5 mAh g^−1^ with the coulombic efficiency stabilized at ~ 100%. For the pristine SiC anode, although the cycling capacity also reveals the good reversibility after 200 cycles, the specific capacity is only 341.6 mAh g^−1^ at 0.1 A g^−1^ after 200 cycles, much lower than that of the NG@SiC anode. Therefore, the inert SiC can be activated by high-quality epitaxial NG layer via enhanced electronic conductivity and accelerated lithium-ion intercalation reaction.

Compared with the pristine SiC anode, the rate analysis (Fig. 4d) reveals that the NG@SiC anode demonstrates a superior high-rate capability from 0.1 to 10.0 A g^−1^, resulting from the improved interatomic electron migration and charge transfer kinetics after the epitaxial NG layer growth. More specifically, the NG@SiC anode delivers stable capacities of 1189.3, 1067.5, 978.2, 886.4, 762.3, 651.5, and 437.1 mAh g^−1^ from 0.1 to 10.0 A g^−1^, showing the excellent high-rate capability in comparison to that of the pristine SiC anode. After cycled at a high-current density of 10.0 A g^−1^, the specific capacity of the NG@SiC anode can maintain ~ 36.7% of the discharge capacity at 0.1 A g^−1^. Despite cycling at a high-current density, the discharge capacity can well recover after the current density drops back to 0.1 A g^−1^. The high-quality epitaxial NG layer can suppress the aggregation of NG@SiC particles and expose abundant active sites for lithium-ion storage. After the epitaxial NG layer growth, sufficient electron/ion bridges were constructed for interatomic electron migration. Thus, the high-inter-particle electronic conductivity and intrinsic electric field can significantly accelerate the diffusion kinetics of lithiation and delithiation. The good lithium-ion storage reversibility and high-rate performance of the NG@SiC anode can be attributed to the intensive interfacial interactions and intrinsic electric field. In Fig. 4f, the comparison of the discharge capacity, capacity retention ratio, and cycle number with the existing Si-based anodes reveals the good high-rate capability and electrochemical reversibility of the NG@SiC anode [10, 12, 17, 45–48]. The more detailed comparison between the NG@SiC anode and other reported Si-based anodes is shown in Table S1. The NG@SiC anode exhibits a better cycling capability than those of the existing Si-based anodes. This comparative analysis shows that interfacial interaction strategy accelerated interfacial charge migration open up an opportunity to design high-performance anode materials, which is important in enhancing the lithium-ion transfer kinetics.

The enhanced electronic conductivity and lithium-ion diffusion kinetics after the epitaxial NG layer growth are further investigated by electrochemical impedance spectroscopy (EIS) measurements from 100 kHz to 0.01 Hz (Fig. 4e). For both the pristine SiC and NG@SiC, the Nyquist plots in the high-to-medium frequency region and the low frequency region consist of semicircles and straight lines, respectively. The high-to-medium frequency region shows the charge transfer resistance (*R*_*ct*_) near the interface, and the low frequency region shows the lithium-ion diffusion resistance (*R*_w_). After fitting with the equivalent circuit model (Fig. S1), the smaller diameter in the high-to-medium frequency region and the larger slope in the low frequency region further confirm the higher electronic conductivity and lithium-ion transfer kinetics of the NG@SiC anode. This result reveals that the epitaxial NG layer can enhance the charge transfer kinetics and interfacial electron density for superior electrochemical performance compared with the pristine SiC. The lithium-ion diffusion coefficient (*D*_Li_^+^) can be obtained from the Nyquist plots via Eq. (1):

1 $$D_{{{\text{Li}}}}^{ + } = R^{2} T^{2} / \, 2A^{2} n^{4} F^{4} C^{2} \sigma^{2}$$

where *C* is the lithium-ion molar concentration, *F* is the Faraday constant, *A* is the surface area of the NG@SiC anode,* T* is the absolute temperature, and *R* is the gas constant. Warburg factor (*σ*) is calculated via Eq. (2):

2 $$Z_{{{\text{real}}}} = R_{e} + R_{{{\text{ct}}}} + \sigma \omega^{ - 1/2}$$

where *ω* represents the angular frequency, *R*_ct_ and *R*_*e*_ are the charge transfer resistance and interfacial resistance, respectively. Thus, the values of *σ* can be calculated by linearly fitting *Z*_real_ against *ω*^−1/2^ in the low frequency region. As shown in the inset of Fig. 4e, the linear fitting results show a smaller Warburg factor for the NG@SiC anode, and the calculated lithium-ion diffusion coefficient is approximately 8.9 × 10^−14^ cm^2^ s^−1^, which is much higher than that of the pristine SiC anode (5.2 × 10^−14^ cm^2^ s^−1^). After high-temperature annealing in ammonia atmosphere, the NG layer can be directly formed by sublimation of outer silicon atoms of SiC particles, which can improve the electronic conductivity of the NG@SiC anode by acting as an electron/ion bridge to enhance the electrochemical reaction kinetics. Therefore, the lithium-ion diffusion coefficient of the NG@SiC anode is significantly improved after the high-quality epitaxial NG layer growth. These results are in good agreement with the first-principles calculation analysis in the following discussion.

 Figure 4g shows the long-term cycling performance of the NG@SiC anode at 10.0 A g^−1^. For both the pristine SiC and NG@SiC anodes, the capacity fading is visible at a high-current density, and the lithium-ion storage reaction becomes stable after the activation process. For the NG@SiC anode, the reversible specific capacity can be maintained at about 447.8 mAh g^−1^ after 1000 cycles at 10.0 A g^−1^ with a stabilized coulombic efficiency of nearly 100%, and the capacity retention maintains at about 81.5%. The average capacity fading per cycle is about 0.018%. For the SiC anode, the specific capacity is about 203.4 mAh g^−1^ after 1000 cycles at 10.0 A g^−1^, which is much lower than that of the NG@SiC anode. The average capacity fading per cycle is about 0.032%. Therefore, the prolonged cycling stability of the NG@SiC anode is significantly improved after the epitaxial NG layer growth, revealing the good reversibility and lithium-ion insertion kinetics of the NG@SiC anode. Compared with the pristine SiC anode, the high-quality epitaxial NG layer can activate the inert SiC with enhanced electronic conductivity and accelerated lithium-ion intercalation reaction. The long-term cycling performance also confirms the good electrochemical reversibility of the NG@SiC anode. After 1000 cycles, the surface morphology change of the NG@SiC anode is shown in Fig. S2, where no particle pulverization or crack can be observed after the reaction at a high-current density, showing the outstanding structural integrity of the NG@SiC anode after long-term (de)lithiation reactions. The excellent high-rate capability and long-term cycling stability of the NG@SiC anode might be ascribed to the synergistic effects of the compositional and structural benefits. First, N doping can enhance electronic conductivity and provide abundant charge storage sites for the NG@SiC anode. Second, the good inter-particle electronic conductivity offered by the epitaxial NG layer can enhance the charge transfer kinetics and reduce the energy barrier of lithium-ion diffusion. Third, the epitaxial NG layer coated structure ensures sufficient access of the electrolyte and lithium-ion intercalation in the NG@SiC anode. Fourth, the excellent structural and chemical stability can suppress side reactions and prevent pulverization/aggregation during the electrochemical reaction.

### Electrochemical Kinetic Analysis

**Fig. 5:**
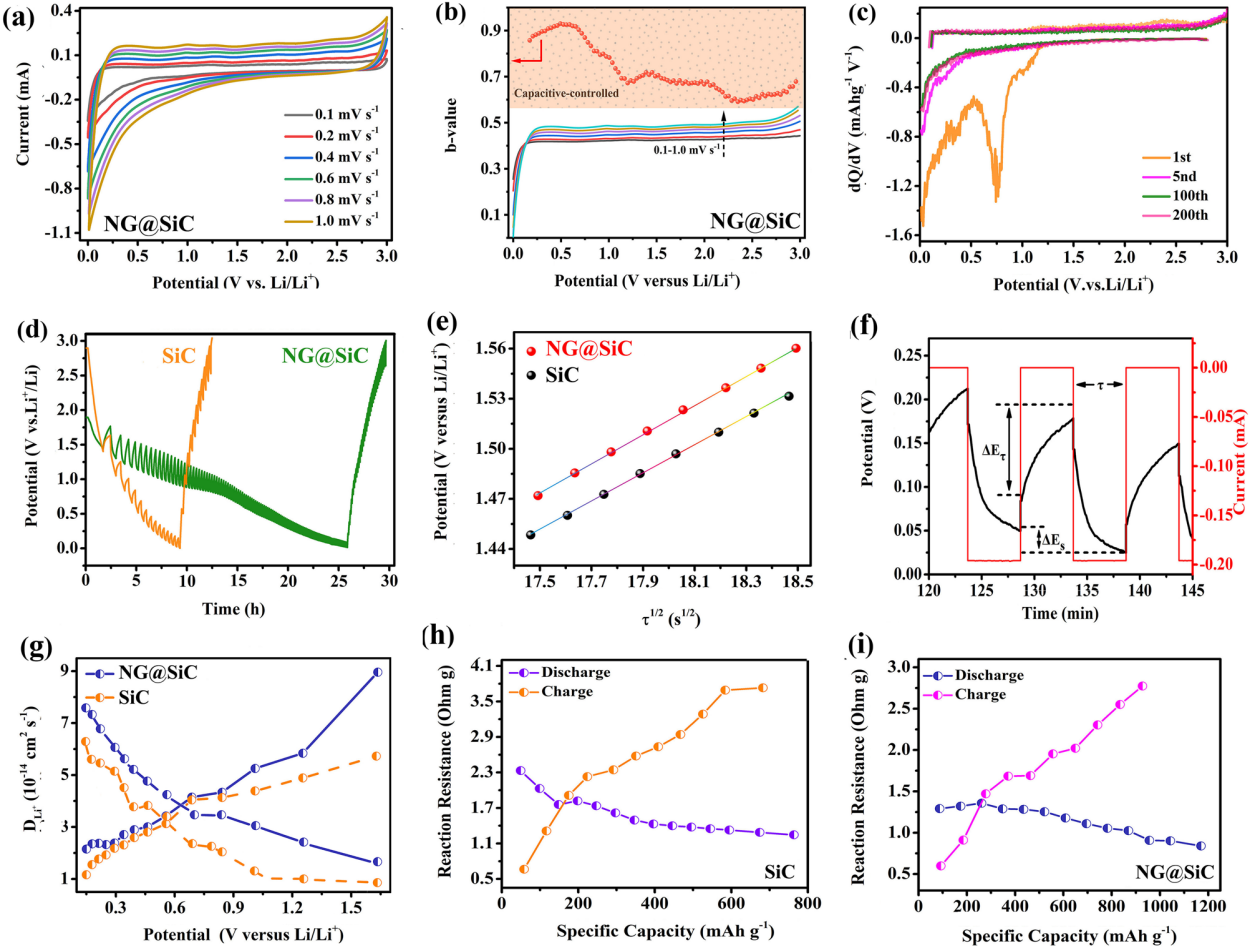
Electrochemical kinetics analysis: **a** CV curves at different scan rate. **b** Calculated b values based on CV curves. **c** dQ/dV curves at different selected cycles. **d** Galvanostatic intermittent titration technique (GITT) profiles. **e** The relationship between potential and τ^1/2^. **f** Illustrated titration profile of GITT. **g** Lithium-ion diffusion coefficient calculated based on GITT. **h, i** Reaction resistances of pristine SiC anode and NG@SiC anode

The electrochemical reaction kinetics was analyzed to understand the high-rate capability of the NG@SiC anode after the high-quality epitaxial NG layer growth. As shown in Fig. 5a, CV tests were carried out from 0.1 to 1.0 mV s^−1^ to quantitatively analyze the pseudo-capacitance contribution. During the continuous lithiation and delithiation reactions, the good reproducibility of the CV curves at different scan rates reveals the stable lithium-ion storage property [49], and the well-defined CV curves indicate that the lithiation and delithiation reactions do not change with different scan rates [50]. The total lithium-ion storage capacity includes the capacitive capacity and Faradaic capacity. The capacitive capacity on the surface and the Faradaic capacity in the bulk can be qualitatively calculated based on Eq. (3) [51]:

3 $$i = av^{b}$$

where *i* is the current density,* v* is the sweep rate, *a* and *b* are adjustable parameters. The *b* values are 0.5 and 1.0 for the Faradaic diffusion process and the capacitive process, respectively [52]. As shown in Fig. 5b, the calculated *b* value is between 0.5 and 1.0. This result reveals that the total lithium-ion storage capacity of NG@SiC includes both capacitive capacity and Faradaic capacity. Figure 5c demonstrates the differential capacity (dQ/dV) curves of NG@SiC during the first, fifth, 100th and 200th cycles. The apparent peak at 0.61 V in the first discharge dQ/dV curve is related to the SEI film, consistent with the CV analysis and previous report. The nearly overlapped dQ/dV curves of the subsequent fifth, 100 and 200th cycles indicate the stabilized lithium-ion intercalation and de-intercalation reaction and good structural reversibility of NG@SiC.

Galvanostatic intermittent titration technique (GITT) measurements were further conducted to calculate the lithium-ion diffusion rate of the NG@SiC anode after the epitaxial NG growth. The GITT analysis was performed after the first cycle to eliminate the interference from the side reactions in the first cycle and reflect the intrinsic characteristics of the electrode materials. The GITT curves of the pristine SiC and NG@SiC do not show obvious discharge or charge plateau during the electrochemical reaction (Fig. 5d), which agrees well with the GCD and CV results. Compared with the pristine SiC anode, the NG@SiC anode shows a higher discharge capacity and a lower polarization, which can be attributed to the intensive interfacial interactions and enhanced lithium-ion diffusion kinetics after the epitaxial NG growth. In addition, the lower overpotential of the NG@SiC anode reveals the faster charge transfer kinetics of the (de)lithiation processes [53]. As shown in Fig. 5e, *E*_*τ*_ is linearly proportional to *τ*^1/2^, and the lithium-ion solid-state diffusion coefficient (*D*_Li_^+^) can be quantitatively determined using Eq. (4):

4 $$D_{{{\text{Li}}}}^{ + } = \frac{4}{\pi \tau }\left( {\frac{{m_{B} V_{B} }}{{M_{B} S}}} \right)^{2} \left( {\frac{{\Delta E_{s} }}{{\Delta E_{\tau } }}} \right)^{2} \left( {\tau \ll \frac{{L^{2} }}{{D_{{{\text{Li}}^{ + } }} }}} \right)$$

where *V*_*B*_, *m*_*B*_, and *M*_*B*_ are the molar volume, the active mass of the electrode, and the molecular weight, respectively. *S* is the contact area between the electrode and the electrolyte,* L* is the thickness of the NG@SiC anode, and *τ* is the intermittent time. As illustrated in Fig. 5f, Δ*E*_*τ*_ (V) is the voltage change through a current pulse, and Δ*E*_*s*_ (V) is the steady-state voltage change. In Fig. 5g, the calculated *D*_Li_^+^ values at different discharge states show a the similar overall trend for both the pristine SiC and NG@SiC anodes, revealing the unchanged (de)lithiation mechanisms and lithium-ion diffusion behaviors after the epitaxial NG growth. The calculated *D*_*Li*_^+^ values of the NG@SiC anode are in the range of 10^−14^ cm^2^ s^−1^, which is higher than those of the pristine SiC anode. This result shows the higher lithium-ion diffusion kinetics and lower polarization with intensive interfacial interactions in the NG@SiC anode [54]. Compared with the pristine SiC, the larger *D*_Li_^+^ values indicate the faster electrochemical reaction kinetics, which is consistent with the EIS analysis and first-principles calculation analysis in the following discussion. Owing to the enhanced electrochemical reaction kinetics after the epitaxial NG growth, the NG@SiC anode delivers higher discharge capacity and rate capability with reduced capacity fading, superior to the pristine SiC anode. Because of the nearly quasi-equilibrium condition, lithium ions have sufficient time to react with the pristine SiC and NG@SiC. Therefore, the reaction resistances at different (de)lithiation states were calculated based on the pulse current, quasi-open-circuit voltage (QOCV), and closed-circuit voltage (CCV) [55]. In Fig. 5h, i, the reaction resistances for both the pristine SiC and NG@SiC anodes gradually decrease during the lithiation process and gradually increase during the delithiation process. Compared with the pristine SiC anode, the reaction resistances and overpotentials of the NG@SiC anode are reduced, showing the enhanced electronic conductivity and accelerated lithium-ion intercalation kinetics of the NG@SiC anode. This result can be attributed to the enhanced interfacial interactions and interatomic electron migrations with a high-surface electron density after the epitaxial NG growth, which is consistent with the first-principles calculation analysis in the following discussion.

### Density Functional Theory Analysis

**Fig. 6:**
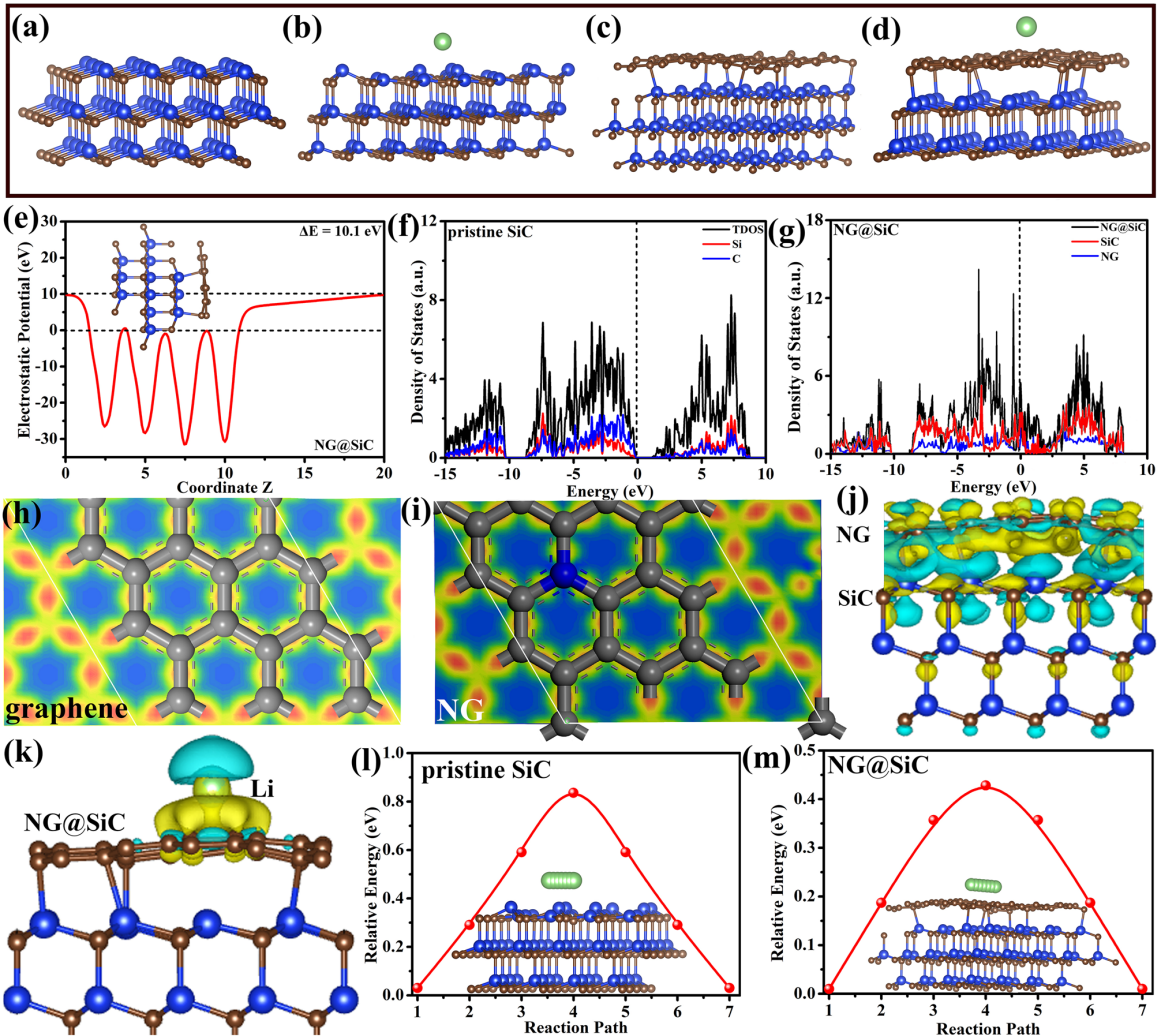
**a-d** Structure illustration of pristine SiC and NG@SiC before and after lithium-ion adsorption, respectively. **e** Electrostatic potential of NG@SiC. **f, g** Density of states (DOS) of the pristine SiC and NG@SiC. **h, i** Charge density difference of graphene and NG. **j, k** Charge density distribution with charge depletion (cyan) and accumulation (yellow) in NG@SiC before and after lithium-ion adsorption. **l, m** Calculated lithium-ion diffusion barrier in pristine SiC and NG@SiC. Inset: corresponding lithium-ion diffusion pathway

Density functional theory (DFT) calculations were employed to further analyze the interfacial electronic structure and to reveal the origin of the outstanding lithium-ion storage performance before and after the epitaxial NG layer growth at the atomic scale. As shown in Fig. 6a-d, the optimized structures of the pristine SiC and NG@SiC before and after lithium-ion adsorption were constructed based on the aforementioned experimental analysis. After the epitaxial NG layer growth and lithium-ion adsorption, the crystal structure of NG@SiC remains the same. The strong interfacial bonding between NG and SiC is distinctly observed after structural optimization (Fig. 6c). This optimized structure also indicates that the epitaxial NG layer is chemically connected to the surface of SiC particles after sublimation of silicon atoms under high-temperature treatment, which has been accurately confirmed by the structural analysis in Fig. 3 (XPS and XAFS). Conceptually, the interfacial electron migration and electrochemical kinetics have great influence on the lithium-ion storage performance of the NG@SiC anode. Therefore, we first estimated the electrostatic potential variation and interfacial electronic transfer in NG@SiC. As shown in Fig. 6e, the planar electrostatic potential result clearly shows a distinct planar electrostatic potential variation at the interface of NG@SiC. The planar electrostatic potential on the NG side is much higher than that on the SiC side, and the difference is about 10.1 eV. Thus, the NG side can accumulate electrons with charge redistribution near the interface, which agrees well with the Si and C *K*-edge analysis. This planar electrostatic potential result demonstrates that the intrinsic electric field is constructed at the interface of NG@SiC, which is beneficial to the interatomic electron migration and charge transfer. The discontinuous potential on the SiC side can be attributed to the dipole moment correction, and the electrostatic potential result is consistent well with the electronic conductivity and EIS analysis. Moreover, the density of states (DOS) of the pristine SiC and NG@SiC was analyzed. In Fig. 6f, the calculated TDOS result shows that the pristine SiC is a direct band gap semiconductor with discrete electronic state, and the calculated PDOSs of Si and C also confirm this analysis (Fig. S3). In Fig. 6g, the calculated TDOS result shows that the electronic state of NG overlap with the hybridized electronic state near the Fermi level. Therefore, the NG@SiC anode possesses a richer electronic state and distinct interfacial interactions for electron transfer, and the distinct interfacial interactions primarily come from the strong covalent bonding. The richer electronic state near the Fermi level shows higher electronic conductivity and carrier density of NG@SiC, which can improve the chemical activity for lithium-ion absorption during the electrochemical process [56]. This electronic state result agrees well with the planar electrostatic potential analysis. The richer electronic state near the Fermi Level contributes to the decreased occupation of anti-bonding states and the enhanced lithium-ion adsorption, based on the orbital hybridization analysis. Therefore, this enhanced electronic state is essential to the fast electrochemical reaction kinetics of the NG@SiC anode.

To further investigate the interfacial interactions at the interface of NG@SiC, charge density difference analysis was performed. Figure 6h-i show the optimized structures of graphene and NG. After N doping, the crystal structure of NG remains similar to that of graphene with slight lattice distortion, and the C atoms near the N atom are electronically perturbed. The charge density difference analysis shows electron depletion near the N atoms and electron accumulation near the C atoms (Fig. 6i), indicating that the electrons transfer from N to C atoms. Therefore, N doping related charge polarization can enrich the electron density of the high-quality epitaxial NG with a better electrochemical activity. Compared with the pristine C atoms (Fig. 6h), the electron depletion region becomes darker for NG (Fig. 6i), indicating the apparent electron depletion and electric field in the high-quality epitaxial NG after high-temperature annealing in ammonia atmosphere. Moreover, the charge density difference of the NG@SiC anode was also calculated. As shown in the 3D isosurface in Fig. 6j, the charge density redistributes at the interface between NG and SiC, and the charge accumulates near the NG side with distinct interfacial interactions, beneficial to the electrochemical reaction kinetics of the NG@SiC anode. Bader charge analysis shows that 0.36 e^−^ is transferred from SiC to NG, indicating the improved electron mobility at the interface of NG@SiC after the quasi-freestanding epitaxial EG was formed. This charge redistribution can prominently regulate the electronic structure and enhance the lithium-ion adsorption at the interface of NG@SiC. Compared with graphene, NG shows a stronger interaction with the adsorbed lithium ion and can regulate the electronic state of SiC on the surface. Therefore, the lithium-ion adsorption energy of NG@SiC (−2.8 eV) is much lower than that of SiC (−1.2 eV), indicating that the epitaxial NG layer can promote the lithium-ion adsorption ability and electrochemical reaction kinetics of the NG@SiC anode during the cycling process [57]. In Fig. 6k, charge density distribution analysis after lithium-ion adsorption demonstrates the charge transfer from the lithium ion to NG@SiC, and the charge accumulation intensity of NG@SiC is larger than that of the pristine SiC (Figs. S4 and S5). This result reveals that the NG@SiC heterojunction shows distinct interfacial binding with lithium ions, resulting in the enhanced adsorption energy and specific capacity. DFT analysis was further performed to understand the migration behavior of lithium ions. The inset of Fig. 6l shows the diffusion path of a lithium ion on the pristine SiC, and the calculated diffusion energy barrier is 0.84 eV (Fig. 6l). After the formation of the epitaxial graphene, the calculated lithium-ion diffusion barrier on NG@SiC drops to 0.43 eV (Fig. 6m), showing the lowered diffusion barrier and enhanced charge transfer kinetics in NG@SiC. This diffusion barrier analysis shows that the epitaxial NG layer has significant impacts on the electrochemical reaction kinetics.

### LiFePO_4_/C//NG@SiC Full Cell Performance

**Fig. 7:**
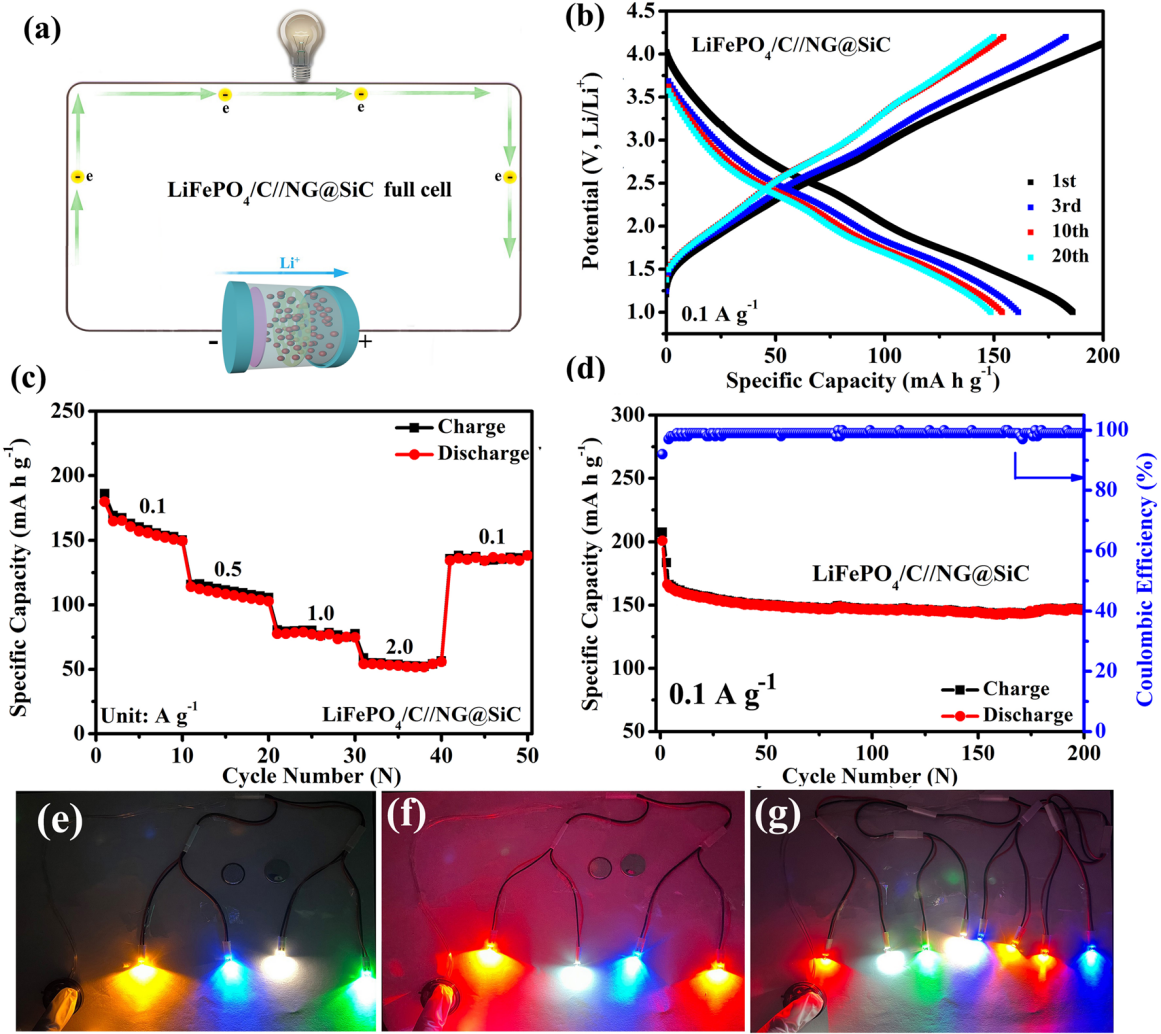
The electrochemical performance of the LiFePO_4_/C//NG@SiC full cell. **a** Schematic illustration of the full cell configuration. **b** GCD curves at 0.1 A g^−1^. **c** Rate capability from 0.1 to 2.0 A g^−1^. **d** Cycling performance at 0.1 A g^−1^. **e–g** Different colors LEDs powered by the LiFePO_4_/C//NG@SiC full cell

To further demonstrate the practical applications of NG@SiC anode, a lithium-ion full cell (LiFePO_4_/C//NG@SiC) was fabricated. Because of its high safety and good stability, the commercial LiFePO_4_/C particles were used as the cathode. Thus, the lithium-ion full cell consists of the NG@SiC anode and the LiFePO_4_/C cathode (Fig. 7a). The electrochemical reaction formula of the LiFePO_4_/C//NG@SiC full cell is expressed in Eq. (5):

5 $${\text{LiFePO}}_{{4}} /{\text{C}} + {\text{NG}}@{\text{SiC}} \leftrightarrow {\text{ Li}}_{{{1} - {\text{x}}}} {\text{FePO}}_{{4}} /{\text{C}} + {\text{ Li}}_{{\text{x}}} [{\text{NG}}@{\text{SiC}}]$$

As shown in Fig. S6, the particle size of the LiFePO_4_/C particles is about 1 μm, and the strong diffraction peaks in Fig. S7 (ICDD PDF No. 83–2092) show the good crystallinity of the commercial LiFePO_4_/C particles. The distinct charge–discharge platform (Fig. S8) indicates a stable output voltage at about 3.4 V during the electrochemical reaction. In Fig. S9, the reversible capacity is about 147.6 mAh g^−1^ after 100 cycles, showing the good structural stability of the commercial LiFePO_4_/C particles. The rate capability (Fig. S10) reveals the excellent reversible lithium-ion diffusion kinetics of the commercial LiFePO_4_/C particles. The GCD curves (Fig. 7b) show the stable output energy density, confirming the practical application of the LiFePO_4_/C//NG@SiC full cell at 0.1 A g^−1^. The polarization in the LiFePO_4_/C//NG@SiC full cell performance is attributed to the different thermodynamic paths and mobility gap among the intermediate phase [58]. Similar to the Li metal//NG@SiC half-cell, the rate capability (Fig. 7c) shows the LiFePO_4_/C//NG@SiC full cell demonstrates good rate performance, and the average rate capacities are 152.3, 108.7, 78.9 and 52.3 mAh g^−1^ at 0.1, 0.5, 1.0 and 2.0 A g^−1^, respectively. The rate capability reveals the fast electrochemical reaction kinetics and good structural stability of the LiFePO_4_/C//NG@SiC full cell [59]. The rate capacity can return to 142.6 mAh g^−1^ at 0.1 A g^−1^ after cycling at a high-current density, indicating the good electrochemical reversibility and practicability of the LiFePO_4_/C//NG@SiC full cell. The energy density and power density are estimated based on the total mass of LiFePO_4_ and NG@SiC, and the obtained energy density and power density remain to be 325 Wh kg^−1^ and 216 W kg^−1^ at 0.1 A g^−1^, respectively, which is comparable to the common lithium-ion batteries [60]. This analysis shows that the LiFePO_4_/C//NG@SiC full cell has a competitive practical prospect for high-performance rechargeable batteries. After the epitaxial NG layer growth, the atomic-scale tunable interfacial interactions and interatomic electron migrations are realized, improving the electrochemical reaction kinetics and rate capability of the NG@SiC anode. In Fig. 7d, the cycling performance shows that the discharge capacity is 147.3 mAh g^−1^ at 0.1 A g^−1^ after 200 cycles with a capacity retention of 79.6%, demonstrating the high-electrochemical stability of the LiFePO_4_/C//NG@SiC full cell. Figure 7e, f shows four-different colors LEDs powered by the LiFePO_4_/C//NG@SiC full cell, and Fig. 7g shows the LiFePO_4_/C//NG@SiC full cell can power eight LEDs simultaneously. This result confirms the potential applications of the LiFePO_4_/C//NG@SiC full cell for high-performance energy storage device, and the interfacial interaction tuning strategy can open opportunities to the rational design of SiC-based anodes.

## Conclusions

In conclusion, the atomic-scale NG@SiC with epitaxial NG layer was successfully synthesized by sublimation of silicon atoms on the surface of SiC particles and applied as a low-potential and high-rate intercalation-type anode with good cycling stability for lithium-ion storage. The intensive interfacial interactions with electron/ion bridges were proven by XAFS and XPS results. DFT analysis further confirmed the electronic interactions between NG and SiC. These interfacial interactions play an important role in enhancing the electronic conductivity and electrochemical reaction kinetics of the well-designed NG@SiC anode, which are beneficial to achieving high-rate performance and good cycling stability. As a proof-of-concept study, the assembled LiFePO_4_/C//NG@SiC full cell demonstrates a competitive practical prospect for high-performance rechargeable lithium-ion batteries. This atomic-scale design strategy provides a novel perspective to improve the electrochemical performance of SiC-based nano-architectures and paves the way for the design of high-performance and durable materials for lithium-ion storage.

### Supplementary Information

Below is the link to the electronic supplementary material.

Supplementary file1 (PDF 814 kb)
