# Supplementary material for: High-Quality Epitaxial N Doped Graphene on SiC with Tunable Interfacial Interactions via Electron/Ion Bridges for Stable Lithium-Ion Storage
Source: Nanomicro Lett. 2023 Aug 18;15:202. doi: 10.1007/s40820-023-01175-6 (PMC10439101; doi:10.1007/s40820-023-01175-6)
Supplement: Supplementary file 1 — Supplementary file1 (PDF 814 kb) [file 40820_2023_1175_MOESM1_ESM.pdf]

Supporting Information for

High-Quality Epitaxial N Doped Graphene on SiC with Tunable Interfacial Interactions via Electron/Ion Bridges for Stable Lithium-Ion Storage

Changlong Sun[1], Xin Xu[1], Cenlin Gui[1], Fuzhou Chen[1], Yian Wang[2], Shengzhou Chen[1], Minhua Shao[2,] *, Jiahai Wang[1,] *

[1] School of Chemistry and Chemical Engineering, Guangzhou University, Guangzhou 510006, P. R. China

[2] Department of Chemical and Biological Engineering, The Hong Kong University of Science and Technology, Clear Water Bay, Kowloon, Hong Kong, P. R. China

*Corresponding authors. E-mail: jiahaiwang@gzhu.edu.cn (Jiahai Wang), kemshao@ust.hk (Minhua Shao)

Supplementary Figures and Tables

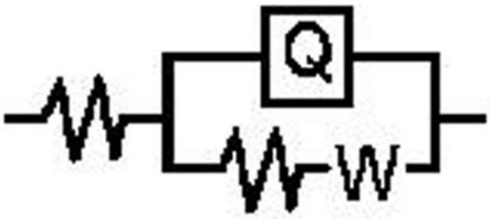

Fig. S1 Equivalent circuit model of the EIS spectrum

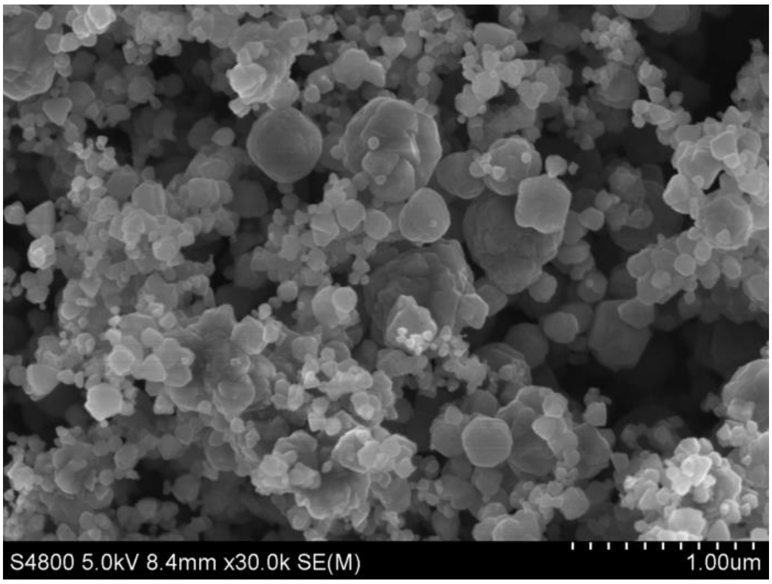

Fig. S2 Morphology change of NG@SiC particles after 1000 cyclesAfter 1000 cycles

at 10.0 A g^{-1}, the SEM image of the NG@SiC particles does not show particle pulverization or crack can be observed after high current density reaction, revealing the outstanding structural integrity after long-term lithiation and delithiation reactions.

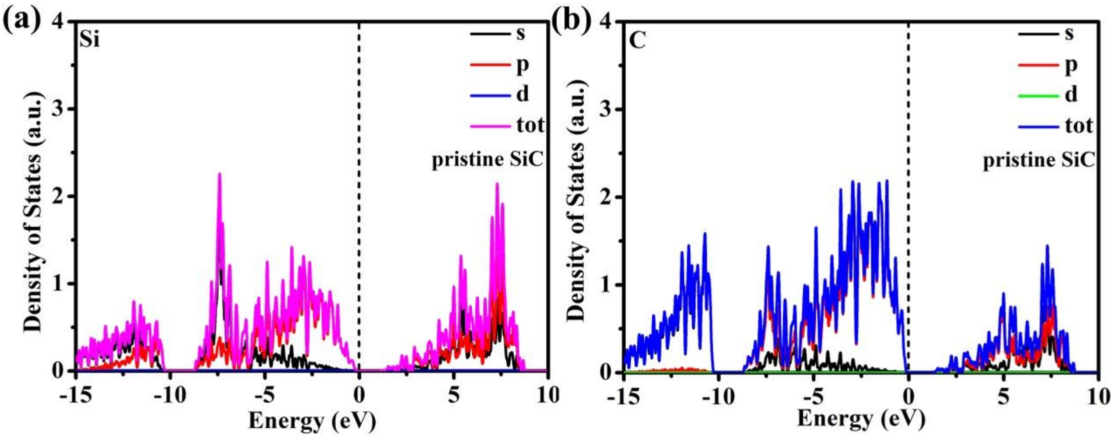

Fig. S3 Partial density of states (PDOS) of the Si and C in pristine SiC.The calculated TDOS result shows that the pristine SiC is direct band gap semiconductor with discrete electronic state, and the calculated PDOS of Si and C in pristine Si also confirm this result.

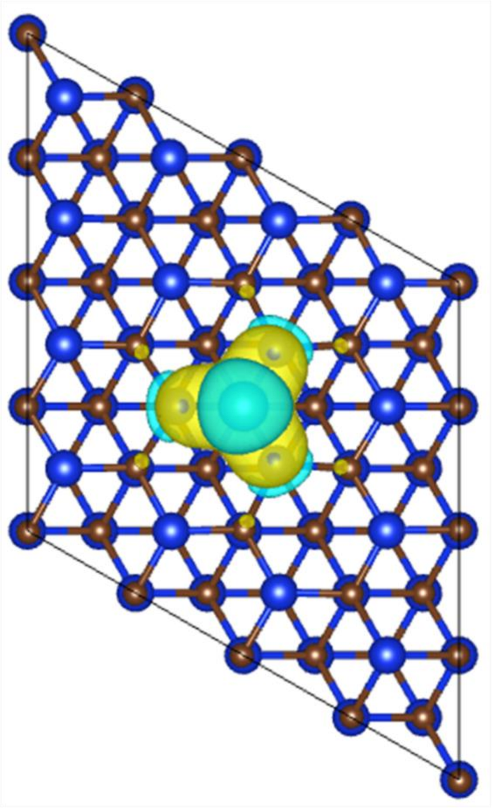

Fig. S4 Charge density distribution of pristine SiC after lithium-ion adsorption

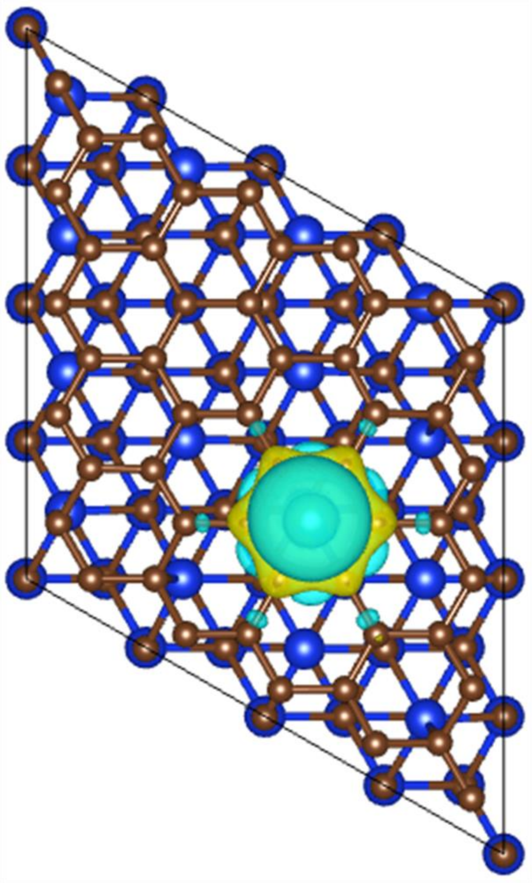

Fig. S5 Charge density distribution of NG@ SiC after lithium-ion adsorptionCharge density distribution analysis after lithium-ion adsorption demonstrate the charge transfer from lithium ion to NG@SiC, and the charge accumulation intensity is larger than that of pristine SiC.

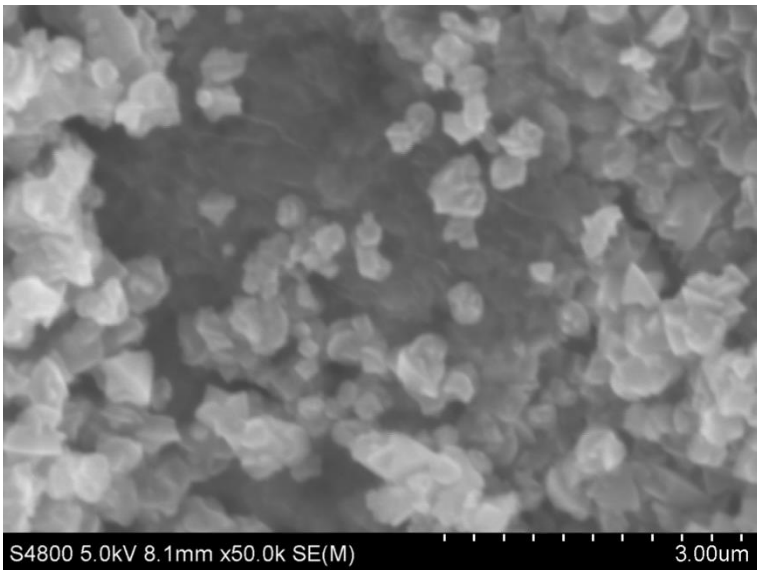

Fig. S6 SEM image of the commercialized $LiFePO_4$/C cathodeAs shown in Figure S6, it can be seen that the commercial $LiFePO_4$/C is composed of irregular particles with the particle size of about 1.0 μm.

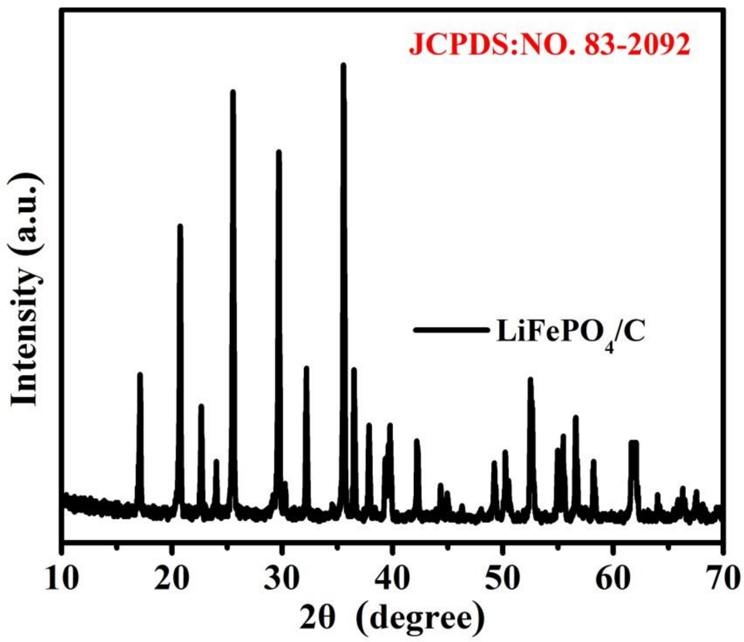

Fig. S7 XRD pattern of the commercialized $LiFePO_4/C$ cathodeThe strong diffraction peaks show the good crystallinity of the commercial $LiFePO_4/C$ particles (ICDD PDF no. 83-2092)

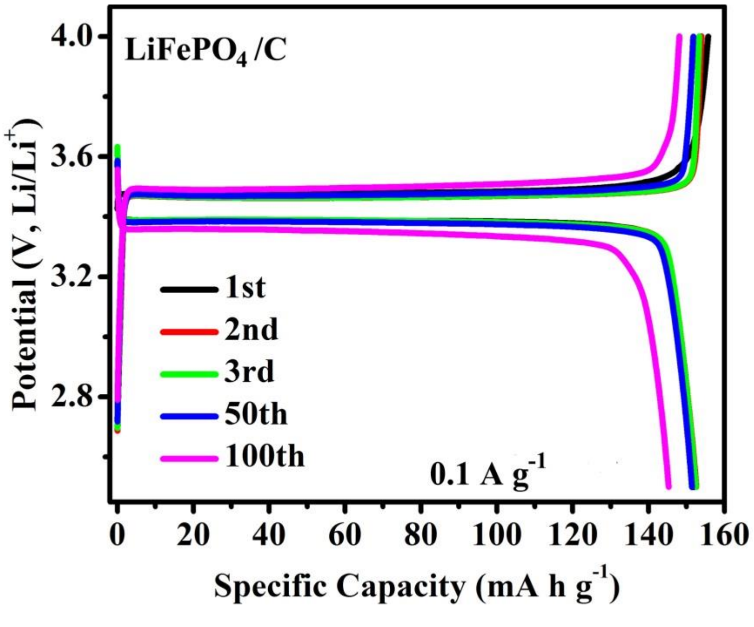

Fig. S8 Charge and discharge curves of the commercialized $LiFePO_4/C$ cathode at 0.1 A g^{-1} of the initial three cycles.

The initial discharge and charge capacities of the commercialized $LiFePO_4/C$ cathode are 152.4 and 151.8 mA h g^{-1}, respectively. The distinct charge-discharge platform indicates stable output voltage at about 3.4 V during the electrochemical reaction.

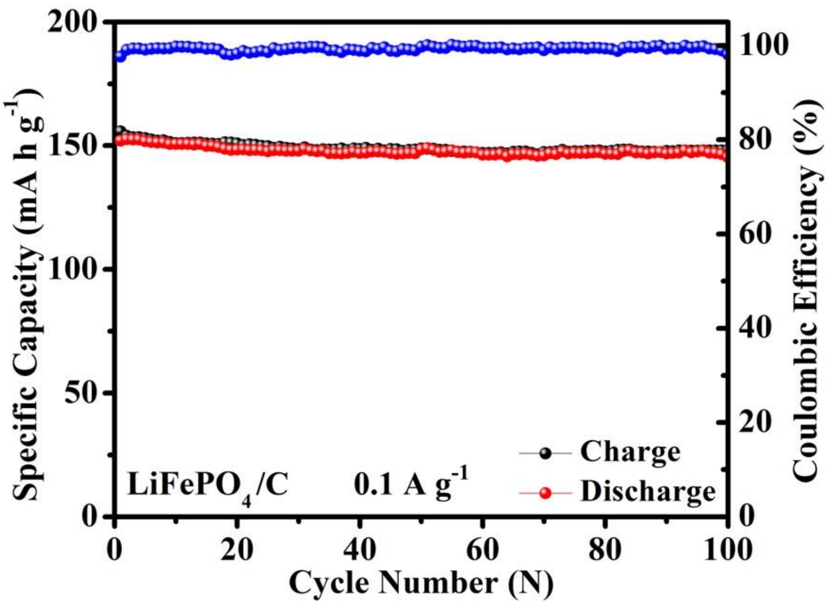

Fig. S9 Cycling stability at 0.1 A g^{-1} of the commercialized $LiFePO_4$/C cathode in the Li-ion half cells for 100 cyclesAfter 100 cycles, the reversible capacity is about 148.6 mAh g^{-1} after 100 cycles, and the Coulombic efficiency can stabilize at ~100%, showing good structural stability of the commercial $LiFePO_4$/C particles.

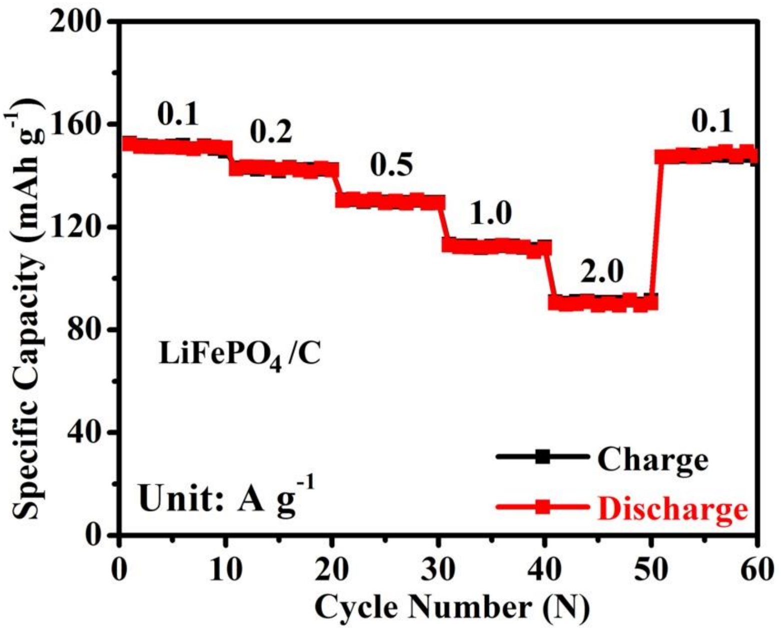

Fig. S10 Rate performance of the commercialized $LiFePO_4$/C at various current densitiesThe commercialized $LiFePO_4$/C cathode can deliver the reversible capacities of 151.8, 141.3, 129.7, 112.9, and 89.4 mA h g^{-1} at 0.1, 0.2, 0.5, 1.0, 2.0 and A g^{-1}, respectively, revealing the good structural tolerance and lithium-ion storage reversibility of the commercialized $LiFePO_4$/C cathode.

Table S1 Comparisons of the synthetic method, morphology, cycle number, current density, and capacity between NG@SiC anode and other previously reported Si-based LIBs anodes

**Material**	**Method**	**Morphology**	**Current density (A g^{-1})**	**Cycle number**	**Capacity (mAh g^{-1})**
**NG@SiC(this work)**	pyrolysis reaction	particles	0.110.0	2001000	1197.5447.8
**SiC@HGSs**[ref.1]	surface graphitization	nanoshells	0.63.0	6001000	1345742
**SiO_2**[ref.2]	mechanical milling	particles	0.5	200	800
**SiO_x/C**[ref.3]	CVD	particles	0.5	500	972
**SiC-Sb-C**[ref.4]	mechanical milling	microspheres	2.0	120	440
**SiC**[ref.5]	ICP-CVD	thin films	0.3 C	100	376
**Si/SiO_x**[ref.6]	direct heating	thin films	0.1	100	1186
**SiO_x/C**[ref.7]	sand milling	particles	0.325	500	645
**Si-O-C**[ref.8]	thermolysis	nanocomposite	1.6	970	200
**SiOC/Sn**[ref.9]	pyrolysis	nanocomposite	0.074	20	562
**SiC/C**[ref.10]	pyrolysis	nanofibers	0.1	250	254.5
**$SiN_{0.92}$**[ref.11]	pulsed laser deposition	thick films	0.02 C	100	700
**SiN_x@Si**[ref.12]	vacuum CVD	nanocomposite	0.5	200	1400
**C@SiO_x**[ref.13]	graphitization	nanospheres	5.0	500	350
**NC@SiO_x**[ref.14]	directly calcining	nanosheets	5.0	1000	427.6
**C@SiO_x**[ref.15]	mixed and heated	hollow spheres	0.51.0	300300	823682
**Si/SiO_2@C**[ref.16]	ball milling	nanoclusters	0.51.0	200200	534.3512.7
**SiOx/G** [ref.17]	calcination	nanocomposite	1.0	1000	780
**SiO_2/TiO_2/C**[ref.18]	annealing	nanocomposite	2.0	400	410
**ZnO-Si@C**[ref.19]	electrospinning	nanofbers	0.81.8	10001000	1050920

Supplementary References

[S1] H. Li, H. Yu, X. Zhang, G. Guo, J. Hu et al., Bowl-like 3C-SiC nanoshells encapsulated in hollow graphitic carbon spheres for high-rate lithium-ion batteries. Chem. Mater. **28**, 1179 (2016). https://doi.org/10.1021/acs.chemmater.5b04750

[S2] W.-S. Chang, C.-M. Park, J.-H. Kim, Y.-U. Kim, G. Jeong et al., Quartz (SiO_2): a new energy storage anode material for Li-ion batteries. Energy Environ. Sci. **5**, 6895 (2012). https://doi.org/10.1039/c2ee00003b

[S3] Z. Liu, Y. Zhao, R. He, W. Luo, J. Meng et al., Yolk@Shell SiOx/C microspheres with semi-graphitic carbon coating on the exterior and interior surfaces for durable lithium storage. Energy Storage Mater. **19**, 299 (2019). https://doi.org/10.1016/j.ensm.2018.10.011

[S4] Z. Chen, Y. Cao, J. Qian, X. Ai, H. Yang, Antimony-coated SiC nanoparticles as

stable and high-capacity anode materials for Li-ion batteries. J. Phys. Chem. C **114**, 15196 (2010). https://doi.org/10.1021/jp104099r

[S5] X.D. Huang, F. Zhang, X.F. Gan, Q.A. Huang, J.Z. Yang et al., Electrochemical characteristics of amorphous silicon carbide film as a lithium-ion battery anode. RSC Adv. **8,** 5189 (2018). https://doi.org/10.1039/C7RA12463E

[S6] W. Yang, H. Liu, Z. Ren, N. Jian, M. Gao et al., A Novel multielement, multiphase, and B-containing SiOx composite as a stable anode material for Li-Ion batteries. Adv. Mater. Interfaces **6**, 1801631 (2019). https://doi.org/10.1002/admi.201801631

[S7] Q. Xu, J.-K. Sun, Y.-X. Yin, Y.-G. Guo, Facile synthesis of blocky SiOx/C with graphite-like structure for high-performance lithium-ion battery anodes. Adv. Funct. Mater. **28**, 1705235 (2018). https://doi.org/10.1002/adfm.201705235

[S8] L. David, R. Bhandavat, U. Barrera, G. Singh, Silicon oxycarbide glass-graphene composite paper electrode for long-cycle lithium-ion batteries. Nat. Commun. **7**, 10998 (2016). https://doi.org/10.1038/ncomms10998

[S9] J. Kaspar, C. Terzioglu, E. Ionescu, M. Graczyk-Zajac, S. Hapis, H.-J. Kleebe, R. Riedel, Stable SiOC/Sn nanocomposite anodes for lithium-ion batteries with outstanding cycling stability. Adv. Funct. Mater. **24**, 4097 (2014). https://doi.org/10.1002/adfm.201303828

[S10] X. Sun, C. Shao, F. Zhang, Y. Li, Q.-H. Wu et al., SiC nanofibers as long-life lithium-ion battery anode materials. Front. Chem. **6,** 166 (2018). https://doi.org/10.3389/fchem.2018.00166

[S11] N. Suzuki, R.B. Cervera, T. Ohnishi, K. Takada, Silicon nitride thin film electrode for lithium-ion batteries. J. Power Sources **231**, 186 (2013). https://doi.org/10.1016/j.jpowsour.2012.12.097

[S12] R.C. de Guzman, J. Yang, M. Ming-Cheng Cheng, S.O. Salley, K.Y.S. Ng, High capacity silicon nitride-based composite anodes for lithium ion batteries. J. Mater. Chem. A **2**, 14577 (2014). https://doi.org/10.1039/C4TA02596B

[S13] G. Zhu, F. Zhang, X. Li, W. Luo, L. Li et al., Engineering the distribution of carbon in silicon oxide nanospheres at the atomic level for highly stable anodes. Angew. Chem. Int. Ed. **58**, 6669 (2019). https://doi.org/10.1002/anie.201902083

[S14] X. Guo, Y.-Z. Zhang, F. Zhang, Q. Li, D.H. Anjum et al., A novel strategy for the synthesis of highly stable ternary SiOx composites for Li-ion-battery anodes. J. Mater. Chem. A **7**, 15969 (2019). https://doi.org/10.1039/C9TA04062E

[S15] T. Xu, Q. Wang, J. Zhang, X. Xie, B. Xia, Green Synthesis of Dual Carbon Conductive Network-Encapsulated Hollow SiOx Spheres for Superior Lithium-Ion Batteries. ACS Appl. Mater. Interfaces **11**, 19959 (2019). https://doi.org/10.1021/acsami.9b03070

[S16] M. Cui, L. Wang, X. Guo, E. Wang, Y. Yang et al., Designing of hierarchical mesoporous/macroporous silicon-based composite anode material for low-cost high-performance lithium-ion batteries. J. Mater. Chem. A **7**, 3874 (2019).

https://doi.org/10.1039/C8TA11684A
[S17] Q. Xu, J.-K. Sun, Z.-L. Yu, Y.-X. Yin, S. Xin et al., SiOx Encapsulated in graphene bubble film: an ultrastable li-ion battery anode. Adv. Mater. **30**, 1707430 (2018). https://doi.org/10.1002/adma.201707430
[S18] L. Zhang, X. Gu, C. Yan, S. Zhang, L. Li et al., Reactive B/Ti nano-multilayers with superior performance in plasma generation. ACS Appl. Mater. Interfaces **10**, 44463 (2018). https://doi.org/10.1021/acsami.8b08120
[S19] J. Li, Z. Li, W. Huang, L. Chen, F. Lv et al., A facile strategy to construct silver-modified, ZnO-incorporated and carbon-coated silicon/porous-carbon nanofibers with enhanced lithium storage. Small **15**, 1900436 (2019). https://doi.org/10.1002/smll.201900436
